# Injectable mesoporous bioactive glass/sodium alginate hydrogel loaded with melatonin for intervertebral disc regeneration

**DOI:** 10.1016/j.mtbio.2023.100731

**Published:** 2023-07-17

**Authors:** Ruibang Wu, Leizhen Huang, Qinghong Xia, Zheng Liu, Yong Huang, Yulin Jiang, Juehan Wang, Hong Ding, Ce Zhu, Yueming Song, Limin Liu, Li Zhang, Ganjun Feng

**Affiliations:** a Department of Orthopedic Surgery and Orthopedic Research Institute, West China Hospital, Sichuan University, Chengdu, 610041, Sichuan, China; b Analytical and Testing Center, Sichuan University, Chengdu, 610065, China; c Operating Room of Anesthesia Surgery Center, West China Hospital, Sichuan University / West China School of Nursing, Sichuan University, Chengdu, 610041, China

**Keywords:** Hydrogel, Intervertebral disc, Mechanical properties, Melatonin

## Abstract

Intervertebral disc degeneration (IDD) is a major contributing factor to both lower back and neck pain. As IDD progresses, the intervertebral disc (IVD) loses its ability to maintain its disc height when subjected to axial loading. This failure in the weight-bearing capacity of the IVD is a characteristic feature of degeneration. Natural polymer-based hydrogel, derived from biological polymers, possesses biocompatibility and is able to mimic the structure of extracellular matrix, enabling them to support cellular behavior. However, their mechanical performance is relatively poor, thus limiting their application in IVD regeneration. In this study, we developed an injectable composite hydrogel, namely, Mel-MBG/SA, which is similar to natural weight-bearing IVD. Mesoporous bioactive glasses not only enhance hydrogels, but also act as carriers for melatonin (Mel) to suppress inflammation during IDD. The Mel-MBG/SA hydrogel further provides a mixed system with sustained Mel release to alleviate IL-1β-induced oxidative stress and relieve inflammation associated with IDD pathology. Furthermore, our study shows that this delivery system can effectively suppress inflammation in the rat tail model, which is expected to further promote IVD regeneration. This approach presents a novel strategy for promoting tissue regeneration by effectively modulating the inflammatory environment while harnessing the mechanical properties of the material.

## Graphical abstract

**Figure:**
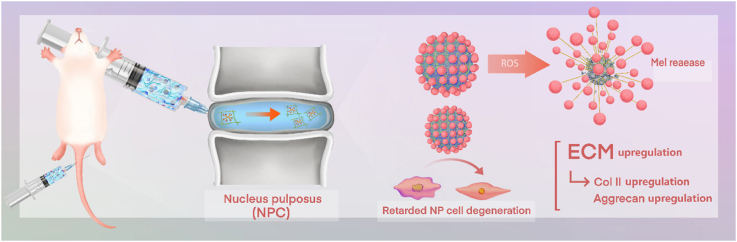

## 1 Introduction

Intervertebral disc (IVD) is an essential component of the spine, situated between adjacent vertebrae. It serves multiple functions, including shock absorption, load distribution, and maintaining spinal flexibility. Anatomically, IVDs consist of the inner nucleus pulposus (NP), the outer annulus fibrosus (AF), and the cartilaginous endplates (CEP). Each of them is vital for maintaining the normal structure and functions of IVD [1]. Intervertebral disc degeneration (IDD) is a prevalent condition that can lead to chronic low back pain and have a significant impact on patients' quality of life. It is a complex and multifactorial disorder influenced by various factors, including aging, injury, genetics, and environmental factors. The consequences of IDD are not only limited to individual patients but also extend to the socioeconomic burden on a global scale [2]. Current conservative treatments for IDD primarily focus on alleviating symptoms and providing pain relief rather than repairing the damaged discs [3]. Invasive surgical treatments, such as spinal fusion, can provide immediate pain relief for some patients with IDD. However, it is important to consider that these surgical interventions do have potential drawbacks and risks. Therefore, researchers have shifted their focus towards developing alternative therapies for IDD, particularly through injecting therapeutics directly into the IVD to slow down the degenerative process and promote disc regeneration [4].

IDD is a complex disease, and the molecular mechanisms underlying its development and progression have not been fully elucidated. Previous research has suggested that IDD is often characterized by inflammatory reactions and decreased extracellular matrix (ECM) in IVD [5]. Inflammatory cytokines such as tumor necrosis factor (TNF), interleukin -1β (IL-1β), and interleukin-6 (IL-6) are known to play critical roles in the progression of IDD. These cytokines can trigger a cascade of inflammatory responses within the disc, leading to various cellular processes that contribute to disc degeneration. A recent study has highlighted the role of oxidative stress in IDD and its association with the inflammatory response and ECM breakdown [6].

Melatonin (Mel), a pleiotropic tryptophan derivative, has been shown to possess antioxidant, anti-inflammatory, and autophagy-regulating properties [7,8]. It is an endogenous molecule found in plants and animals [8], and has been demonstrated to suppress various degenerative disorders including Alzheimer's disease [9], Parkinson’ disease [10], and osteoarthritis [11]. Melatonin exerts its antioxidant effect through a series of scavenging mechanisms. On the one hand, it inhibits the formation of hydroxyl radicals and acts directly as an antioxidant. On the other hand, it stimulates the intracellular antioxidant enzyme system, including the activity of glutathione peroxidase and glutathione reductase, and accelerates the synthesis of glutathione [12]. The activation of two cell membrane melatonin receptors, MT1 and MT2, mediates the biological function of melatonin [13]. Studies have shown that melatonin can downregulate the expression of IL-1β and TNF in human rheumatoid synovial fibroblasts in a dose-dependent manner by interacting with MT1 cell membrane receptors [14]. Furthermore, melatonin has been found to activate nuclear factor erythroid2-related factor 2 (Nrf2) through the MT1/MT2 receptor pathway and alleviate endoplasmic reticulum stress in senescent canine adipose mesenchymal stem cells [15]. These findings suggest that melatonin exerts its anti-inflammatory effects through a pathway mediated by melatonin receptors. Furthermore, melatonin reduces the production of reactive oxygen species (ROS), thereby enhancing cell viability and promoting continuous cell differentiation to protect mesenchymal stem cells (MSCs) from oxidative stress-induced apoptosis [16]. Additionally, there is evidence suggesting that melatonin can induce chondrocyte differentiation via the TGF-β signaling pathway [17]. This beneficially impacts chondrocyte cartilage matrix synthesis, potentially aiding in maintaining healthy cartilage and intervertebral disc [18]. Therefore, we hypothesize that the administration of Mel could potentially halt or even reverse the process of disc inflammation. However, it is worth noting that Mel, when taken orally, has a short half-life and low absorption efficiency. On the other hand, subcutaneous injection of Mel can achieve higher utilization rates, but it is challenging to achieve an effective local concentration [19]. Hence, there is an urgent need to investigate a suitable carrier for loading Mel to enhance its therapeutic effectiveness.

Injectable hydrogels have shown promising results as drug carriers, effectively facilitating local drug delivery while minimizing systemic toxicity [[20], [21], [22]]. These hydrogels have the ability to retain drugs within their three-dimensional network, enabling sustained release and prolonged drug exposure at the desired site. Moreover, hydrogels possess hydration properties similar to those of the nucleus pulposus tissue, making them compatible with the dynamic characteristics of ECM [23]. This makes hydrogels particularly suitable for supporting the survival of nucleus pulposus cells and promoting matrix formation within the IVD [24]. Among them, sodium alginate (SA) hydrogels have emerged as an alternative option for nucleus pulposus tissue repair. SA hydrogels possess characteristics that resemble those of healthy nucleus pulposus tissue [25], including their viscoelastic properties and water absorption capacity. They also exhibit excellent biocompatibility and low cytotoxicity, making them suitable for biomedical applications [26]. However, one crucial requirement for an implanted nucleus pulposus substitute is its ability to withstand axial forces and maintain disc height under weight-bearing conditions. The mechanical strength of SA hydrogels alone may not be sufficient to support the compression and weight-bearing demands required for IVD repair [27]. SA hydrogels are known for their soft and deformable nature, which limits their application in scenarios where higher mechanical strength is necessary.

The incorporation of bioactive glass (BG) particles into hydrogels has proven effective in improving their mechanical properties, addressing the issue of insufficient strength [28]. BG is an inorganic material known for its ability to stimulate bone and cartilage regeneration. It has been extensively used in dentistry and orthopedics due to its biocompatibility and regenerative properties [29]. Nanocarriers, such as mesoporous bioactive glass (MBG) nanoparticles, have emerged as a promising approach for drug delivery. These nanoparticles possess a high specific surface area, making it easier to chemically modify their surface and enhance drug delivery efficiency [30]. MBG nanoparticles are widely studied as drug carriers due to their ability to achieve high drug loading and controlled drug release [31]. Previous studies have predominantly focused on the use of simple BG as a material for osteogenesis and bone defect treatment, rather than cartilage repair [[32], [33], [34], [35]]. This is because simple BG shares similar elemental composition with natural bone, releasing various ions that promote osteoblast activity and inhibit osteoclast activity [33]. During the last decade, tissue engineering studies have increasingly explored combination therapies that combine BG with specific agents, such as sodium alginate (SA), to accommodate the different mechanical strength and physiological properties of bone and cartilage [36]. Hydrogels alone often lack the mechanical strength required for bone tissue, while BG alone may be too strong for cartilage and intervertebral disc (IVD) tissue. In cartilage repair, the combination of BG with hydrogels has shown promise in maintaining the cartilage phenotype and enhancing chondrocyte differentiation, while also providing some mechanical strength [37]. On top of this, various factors that promote cartilage regeneration and anti-inflammation, such as ions [38], exosomes [39], and drugs [40], are typically added to the combination of BG and hydrogel to optimize cartilage regeneration. This synergistic strategy, based on enhanced differentiation ability and modulation of the inflammatory response, can also be applied to the IVD, which are cartilage-like tissues. Functionalized hydrogels composed of hydrogels and mesoporous silica nanoparticles have demonstrated the ability to scavenge ROS and promote the secretion of extracellular matrix (ECM) [41]. The presence of MBG in the hydrogel matrix allows for the enhanced administration of the anti-inflammatory and antioxidant effects of melatonin to the IVD environment. More notably, the melatonin-coated hydrogels could inhibit inflammation in the disc, thereby mitigating the progression of disc degeneration.

**Scheme 1:**
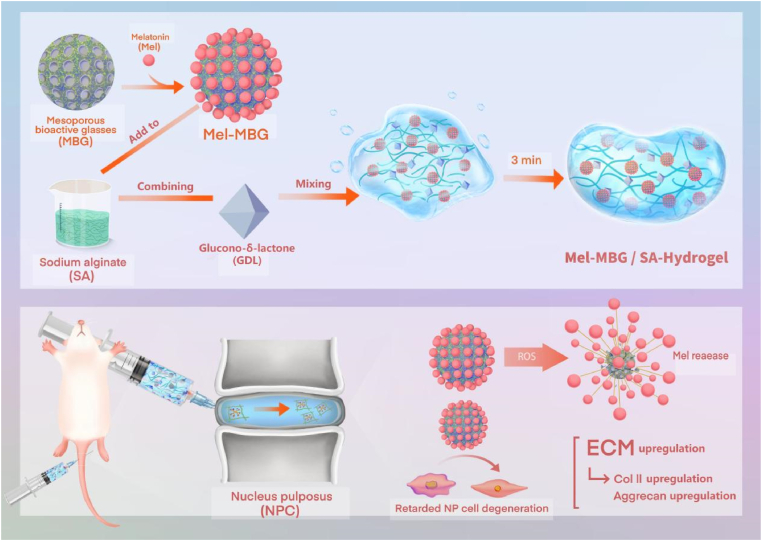
**Schematic diagram for the preparation of injectable MBG-Mel/SA hydrogel.** MBG and Mel were blended and ultra-sonicated for the complete drug loading. The obtained Mel-MBG nanoparticles were incorporated with SA solution, followed by the addition of GDL. Then, the obtained Mel-MBG/SA gel was rapidly injected into the nucleus pulposus region of the rat tail. Under the regulation of GDL, the injected Mel-MBG/SA gel could be converted into solid hydrogel for in situ fixation without displacement.

In this study, a novel injectable composite hydrogel called Mel-MBG/SA was developed. This composite hydrogel aimed to combine the benefits of MBG nanoparticles as a drug carrier and SA hydrogel as a matrix for IVD repair. The composite hydrogel is anticipated to combine the strengths of each component, resulting in a multifunctional material that exhibits several advantageous features. These include injectability, sustainable release of Mel medication, enhanced mechanical strength, biodegradability and biocompatibility. The composite hydrogel exhibits a remarkable ability to conform to the solid-like mechanical properties of the intervertebral disc under high load rates. Notably, the load compression capacity of the composite hydrogel can reach values ranging from 0.75 to 2.75 MPa. The role and underlying mechanism of the composite hydrogel in supporting IVD regeneration were explored by means of cytology, histology, and molecular science. Scheme 1 illustrates the preparation process of Mel-10.13039/100010493MBG/SA composite as an injectable hydrogel for disc regeneration.

## 2 Materials and methods

### 2.1 Materials

Sodium alginate (SA, AR) was purchased from Aladdin Shanghai Co., Ltd. (China). Cetyltrimethylammonium bromide (CTAB, AR, ≥99%) was supplied by Chengdu Kelong Chemical Reagent Co., Ltd. (China). Tetraethyl orthosilicate (TEOS, AR), melatonin (Mel, ≥99%), and glucono-δ-lactone (GDL, AR) were purchased from Sigma-Aldrich (USA). All chemicals used were of analytical grade, and were used as received without any further purification.

### 2.2 Fabrication of Mel-MBG/SA hydrogels

The injectable Mel-MBG/SA hydrogels were prepared according to a previously reported method [42]. Firstly, SA powder (Shanghai Aladdin Biochemical Technology Co., Ltd., China) was completely dissolved in deionized water to obtain a 2% wt SA solution. After filtering through a 0.23-μm filter (Millipore), the solution was stored at 4 °C until further use. MBG was synthesized using CTAB as a template agent according to a previously reported method [43]. The ordered mesoporous structure of the MBG particles was observed by transmission electron microscopy (TEM; Talos F200S, Thermo Fisher, USA). After ultraviolet disinfection, MBG was loaded with Mel for the next step. Briefly, 500 μg/mL Mel (Sigma-Aldrich, USA) was stirred in 1% acetic acid and loaded into different mass fractions of MBG powder. The mass ratios of MBG to Mel were 10:1, 5:1, and 2.5:1. After drying for 24 h, different mass fractions of MBG and Mel mixture (0.5% wt, 1% wt, 2% wt) were added to a 2% wt SA solution, followed by sonication for 30 min and thorough stirring. Then, glucono-δ-lactone (GDL, >99.0%, Sigma-Aldrich) was gradually added during stirring. The inclusion of GDL in the experimental setup created an acidic environment, which facilitated the release of Ca^2+^ ions from MBG. The released Ca^2+^ ions could crosslink SA molecules to form an SA hydrogel at room temperature within a few min. Finally, the resulting hydrogel was stored at 4 °C for 24 h to ensure a complete gelation.

### 2.2 Characterization of Mel-MBG/SA hydrogels

Physico-chemical characterization. The crystallinity of samples was characterized by X-ray diffraction (XRD, DX-2500, Fangyuan, China, Cu Ka radiation) at 40 kV with 25 mA, the scan speed was 2°/min and 2θ range was 10–80°. The compositions were analyzed by a Fourier transform infrared spectroscopy (FT-IR, NICOLET-6700, Thermo, USA). The chemical structure and functional groups of the composite hydrogels were examined by FT-IR in transmission mode at a 4 cm^−1^ resolution and 16 scans with a wavelength range from 500 to 4000 cm^−1^. To confirm the successful incorporation of Mel into the hydrogel, ^13^C NMR spectroscopy was performed. The solutions were examined with a 600 M NMR instrument (Avance III, Burker, Germany) and the spectra were recorded by the device.

To optimize the GDL concentration favorable for SA hydrogel formation, 1 mL of MBG/SA mixture and different concentrations of GDL (10%, 7% wt, 5% wt, 3% wt, 1% wt) were added to small vials and reacted at 37 °C. The vials were tilted every 15 s and inverted every 30 s to observe the state of the mixture until it crosslinked into an immobile gel, and the gel time was recorded. After drying and surface sputter coating with gold, the microstructure of Mel-MBG/SA hydrogels was observed by scanning electron microscopy (SEM, JEOL, JSM 6510 LV, Japan).

To evaluate the swelling behavior and stability of the hydrogel structure, SA hydrogels containing 0.5% wt, 1% wt, and 2% wt MBG were soaked in Simulated Body Fluid (SBF) at a solid-to-liquid ratio of 0.5 g/10 mL. These three hydrogel scaffolds were weighed after freeze-drying overnight to obtain dry weight (Wd). Subsequently, the samples were immersed in SBF at room temperature. At predetermined time points, the samples were extracted and surface water was removed before reweighing to obtain wet weight (Ws). The water uptake was calculated as (Ws/Wd), and the data were recorded as the swelling ratio.

To evaluate the mechanical properties of Mel-MBG/SA hydrogels, the cylindrical hydrogel samples were prepared by molding MBG/SA mixtures containing 0.5% wt, 1% wt, and 2% wt MBG (mold size Φ14 mm × 5 mm). After 24 h of gelation, the resulting hydrogel samples were subjected to compression testing. A universal mechanical testing machine (model E45, USA) was used to evaluate the compressive strength of the hydrogel, with a compression rate of 1 mm/min and a maximum deformation limit of 99%. This method allows us to quantify the force required to deform the hydrogel and the deformation response of the hydrogel as a function of the applied force [44].

### 2.3 *In vitro* Mel releasing behavior

Firstly, the mixtures of 2% wt MBG and Mel at different ratios of 2.5:1, 5:1, and 10:1 were placed in deionized water and ultrasonically blended for 30 min. Next, 2% wt SA powder was added into the above suspension and mixed thoroughly, followed by the manual addition of GDL to obtain the Mel-MBG/SA hydrogel. After 24-h gelation, the hydrogel was immersed in PBS solution and placed in a constant temperature shaking chamber. The absorbance values were measured at different time points (1 h, 3 h, 7 h, 12 h, 24 h, 3 d, 7 d, 12 d, 18 d, and 28 d) using a UV–Vis absorption spectrometer (Lambda25 spectrophotometer, PerkinElmer, USA). Three parallel samples were set up, and the cumulative drug release rate was calculated as follows:

$$Cumulative\;drug\;release\;rate\;\%=\frac{V_{0}\times C_{t}+V\sum_{b=1}^{t-1}C}{m}\times 100\%$$

where c_t_ indicates the concentration of Mel in PBS measured at a certain time point, V denotes the volume of PBS collected at each time point, t represents the time of drug release, c indicates the concentration of Mel in PBS at each time point, and b denotes the release index of the release mechanism.

### 2.4 Preparation of injectable hydrogel extracts

The MBG, GDL, Mel, and SA powders were sterilized under UV light. A sterile solution of SA (2% wt) was then prepared with deionized water. Subsequently, the composite hydrogels were prepared on a clean bench according to the method described in Section 2.2. After gelation, the obtained hydrogels were separately immersed in Dulbecco's Modified Eagle Medium (DMEM; Gibco, USA) at a concentration of 0.1 g/mL, followed by incubation at 37 °C for 24 h. Finally, the resultant solutions were collected and filtered through a 0.22-μm filter, and then stored at 4 °C or −20 °C for long-term preservation.

### 2.5 Cell culture and treatment

The nucleus pulposus cells (NPCs) used in this study were extracted from Sprague-Dawley (SD) rats according to the established procedures. Briefly, the nucleus pulposus tissue of each caudal vertebra was aseptically removed and incubated in type II collagenase (0.25%; Sigma-Aldrich, China) at 37 °C for 2 h. After filtration and centrifugation (1000 rpm, 5 min), the supernatant was collected and resuspended in a 10-cm culture dish containing 8 mL DMEM (low glucose; Gibco, USA) supplemented with 10% FBS (Gibco, USA) and 1% penicillin/streptomycin (Gibco, USA), and then incubated at 37 °C with 5% CO_2_. The medium was changed every 2 days until the cells reached 80% confluence. Passages 3 to 5 were used for subsequent experiments.

### 2.6 Cell viability and DNA content

Prior to conducting cell live-death experiments, a blank group consisting of NP cells cultured in a normal environment was established. The positive control group, 50 μM Mel solution group, and Mel-MBG/SA hydrogel group were subjected to pretreatment with 5 ng/mL IL-1β (Gibco, USA) to induce an inflammatory environment. The biocompatibility of 50 μM Mel solution and Mel-MBG/SA hydrogel was assessed using the Live/Dead Cell Staining Kit (Beyotime, China). NPCs were exposed to medium containing 50 μM Mel or Mel-MBG/SA hydrogel perfusion for 24 h. The cells were washed three times with PBS, and then stained with 2 mM calcein-AM (fluorescent green) and 4 mM propidium iodide (PI, fluorescent red) in PBS for 15 min. After a gentle rinse with PBS, the fluorescence was observed using a fluorescence microscope (Olympus IX83, Japan). Live cells stained with calcein-AM were captured at Ex/Em = 488/517 nm, while dead cells stained with propidium iodide were captured at Ex/Em = 493/636 nm. Cell viability was calculated by dividing the number of live cells by the total number of cells.

The effect of Mel and Mel-MBG/SA extracts on NPC proliferation was evaluated using the Cell Counting Kit-8 (CCK-8; Beyotime, China). The cells were treated with 50 μM Mel and Mel-MBG/SA extracts for 1, 3, and 5 days, and then incubated in medium containing 10% CCK-8 at 37 °C for 2 h. Cell proliferation was determined by measuring the absorbance at 450 nm using a microplate analyzer (Synergy Mx, BioTek, USA). The control group was maintained with normal growth medium.

NPCs were cultured on Mel-MBG/SA hydrogels for 1 d. NPCs were fixed and incubated with appropriate FITC-phalloidin (Beyotime, China) dilutions and DAPI afterwards for cytoskeletal staining and nuclear staining. The fluorescence was observed using a fluorescence microscope (Olympus IX83, Japan).

The fluorescence assay utilizing the QuantiFluo TM DNA assay kit was employed to determine the DNA content in various subgroups. Specifically, on days 1, 3, and 5, trypsin solution was utilized to digest the samples, which were subsequently cooled to room temperature. Standard DNA solutions were prepared through serial dilution, resulting in concentrations of 2000, 1600, 1200, 800, 600, 400, 200, and 0 ng/mL. Following this, 10 μl of both the samples and standards were transferred to a black flat-bottomed 96-well plate, and 100 μl of working reagent was added. After incubation for 1 min at room temperature, the fluorescence signal was measured using a microplate analyzer (Synergy Mx, BioTek, USA) at an excitation wavelength of 346 nm and an emission wavelength of 460 nm.

### 2.7 Cellular immunofluorescence staining

The effect of Mel-MBG/SA extract on the production of ECM in NPCs was evaluated through cellular immunofluorescence using a specific marker for nucleus pulposus (COL2A1). Inflammatory microenvironment was established by supplementing the medium with 5 ng/mL of IL-1β for a specified duration. NPCs were seeded in 6-well plates and treated with normal growth medium or Mel-MBG/SA extract for 3 days. The cells were fixed with 4% paraformaldehyde, permeabilized with 0.1% Triton-X, and blocked with 5% bovine serum albumin (BSA). The cells were then incubated overnight at 4 °C with a primary antibody against COL2A1 (1:250, Abcam, USA), followed by incubation with Alexa Fluor 594-conjugated secondary antibody (Abcam) for 1 h. DAPI staining (Solarbio, China) was performed for 5 min, and the cells were then visualized using a fluorescent microscope (Olympus IX83, Japan).

### 2.8 Cellular ROS detection

To assess the impact of Mel-MBG/SA on reactive oxygen species (ROS) levels in NPCs, the cells were exposed to 5 ng/mL IL-1β (Gibco, USA) and co-cultured with Mel-MBG/SA for 3 days. To measure ROS levels, NPCs were treated with 2′,7′-dichlorofluorescein diacetate (DCFH-DA) (Beyotime, China) at a final concentration of 10 μmol/L at 37 °C for 30 min. After washing the cells three times to remove excess DCFH-DA, the probe-loaded samples were evaluated using a fluorescence microplate reader at 488/525 nm.

### 2.9 RNA isolation and RT-PCR analysis

Total RNA was extracted using TRIzol® reagent (Invitrogen, USA) according to the manufacturer's instructions. iScript cDNA synthesis kit (Bio-Rad) was used to synthesize cDNA from 1 μg of total RNA through reverse transcription, according to the kit's protocol. Real-time PCR was performed with EvaGreen dye (Bio-Rad) using an RT-PCR instrument (CFX Connect, Bio-Rad). After pre-treating NPCs with IL-1β for three days, the culture medium was replaced with Mel-MBG/SA extracts to establish normal control and inflammation control groups. RT-PCR was conducted to detect the mRNA expression levels of COL2A1 (type II collagen) and ACAN (aggrecan) for evaluating the phenotype of NPCs. To observe the anti-inflammatory effects of the materials in each group, the mRNA levels of inflammatory factors and ECM-degrading enzymes (TNF, MMP-3, MMP-13 and ADAMTS-5) were also determined by RT-PCR assay. Glyceraldehyde-3-phosphate dehydrogenase (GAPDH) was employed as an internal reference. The relative expression levels of the target genes were calculated using the comparative Ct (2-ΔΔCt) method. The primer sequences used in this study are listed in Supplementary Table 1.

### 2.10 Western blotting

The present study employed Western-blot analysis to investigate the expression of anabolic/catabolic markers in NP cells treated with IL-1β. Total proteins were extracted from NP cells and subjected to separation by 10% SDS-PAGE gels. The separated proteins were subsequently transferred onto PVDF membranes (Amersham, Buckinghamshire, UK), which were blocked with 5% skim milk in TBST. The membranes were then incubated with primary antibodies overnight at 4 °C. The primary antibodies utilized in this study were anti-aggrecan (ab177480, 1:200; Abcam), anti-mmp3 antibody (ab52915,1:200; Abcam), anti-MMP-13 (ab219620, 1:200; Abcam), anti-ADAMTS-5 (ab41037, 1:200; Abcam), and anti-β-actin (ab8226, 1:1000; Abcam). Following three washes with TBST, the membranes were subjected to incubation with anti-rabbit IgG for 1 h at room temperature. After rinsing with Tris-buffered saline, the membranes were incubated with the secondary antibody for 2 h at room temperature. Finally, proteins were measured by enhanced chemiluminescence using Bio-Rad Image Lab Software 5.2 (Bio-Rad Laboratories, Hercules, CA, USA).

### 2.11 Animal model and histological evaluation

The experimental protocols were approved by the Animal Ethics Committee of West China Hospital, Sichuan University, China (approval reference number: 20230330006), and were conducted in compliance with the guidelines of the Care and Use of Laboratory Animals (China). Twenty-five male Sprague-Dawley (SD) rats (8 weeks old, weighing 200 ± 10 g) were used to establish a rat disc inflammation model by puncturing the tail AF. X-ray was employed to locate the IVDs in Co7/8 (The intervertebral disc located between the seventh and eighth caudal vertebrae of the coccyx). The rats were anesthetized with isoflurane and then disinfected with iodine. Following that, a 20G needle was inserted through the entire annulus fibrosus layer into the center of the experimental layer to induce a puncture injury. All needles were rotated 360° and held in place for 1 min before being removed. One week post-operation, 2 μL of equal amounts of PBS, 50 μM Mel, MBG/SA hydrogels and Mel-MBG/SA hydrogels were injected into the IVDs using 26 G needles. A random assignment of rats into five groups (n = 5 rats/group) was conducted. The group treated with PBS following surgery was designated as the inflammation degeneration control (DC) group (n = 5), while the group without surgical intervention was designated as the normal control (NC) group (n = 5). Radiological evaluations were performed at 4 and 8 weeks post-surgery by obtaining tail vertebrae from the rats for imaging analysis. Digital X-ray fluoroscopy and microcomputed tomography imaging (μCT) were used for the quantitative analysis of IVD height. X-rays of the rat tailbone were taken before and after 4 and 8 weeks post-surgery at the experimental level (Co7/8). The Quantum GX μCT system (PerkinElmer Waltham, USA) was utilized for μCT, employing a voltage of 90 kV and a current of 45 μA, with a slice thickness of 3.0 mm. The disc height index (DHI) was calculated according to a previously published method [45], and the change in DHI was expressed as DHI% (postoperative DHI/preoperative DHI). The 3D and 2D reconstructions were completed using CT Analyse12 software (AnalyzeDirect, Kansas, USA).

MRI evaluations were conducted on the rats at 4 and 8 weeks post-surgery to assess disc inflammation. After anesthetization, MRI was performed using a 7.0 T clinical magnet (BioSpec 70/30, Bruker, USA) to obtain sagittal T2-weighted images, as previously described. The following settings were used for the sagittal T2-weighted images: TR/TE, 3000/40; echo train length, 10; slice thickness, 1 mm; field of view, 8 × 2 cm; and matrix size, 400 × 100.

After an 8-week period, rats were subjected to histological evaluation through execution. The tail vertebrae were extracted and preserved in formalin for 48 h, followed by decalcification in 10% ethylenediaminetetraacetic acid (EDTA) for a duration of 4 weeks. Subsequently, the tail vertebrae underwent continuous dehydration, were embedded in paraffin, and sectioned at a thickness of 3 μm. The sections were then dewaxed and stained using hematoxylin-eosin (HE, Solarbio, China), Safranin O/fast green (Solarbio, China), and Masson trichrome solution (Solarbio, China). The morphology of the intervertebral discs was assessed through microscopic observation and scoring. Subsequently, the sections were subjected to dewaxing and dehydration, followed by incubation with 3% H_2_O_2_ at 37 °C for 10 min. The sections were then washed with PBS for 5 min, boiled in 0.01 M citrate buffer, and blocked with goat serum for 10 min at 37 °C. Primary antibodies (anti-collagen II, 1:200) and (MMP13, 1:200) were applied to the sections overnight at 4 °C, followed by incubation with secondary antibody for 30 min at room temperature.

### 2.12 Statistical analysis

The data are presented as mean ± SD, and each experiment was repeated three times. Statistical differences were determined by one-way analysis of variance (ANOVA). A p-value of *<0.05, **<0.01, and ***<0.001 was considered statistically significant.

## 3 Results

### 3.1 Characterization of MBG and MBG/SA composite hydrogel

**Fig. 1:**
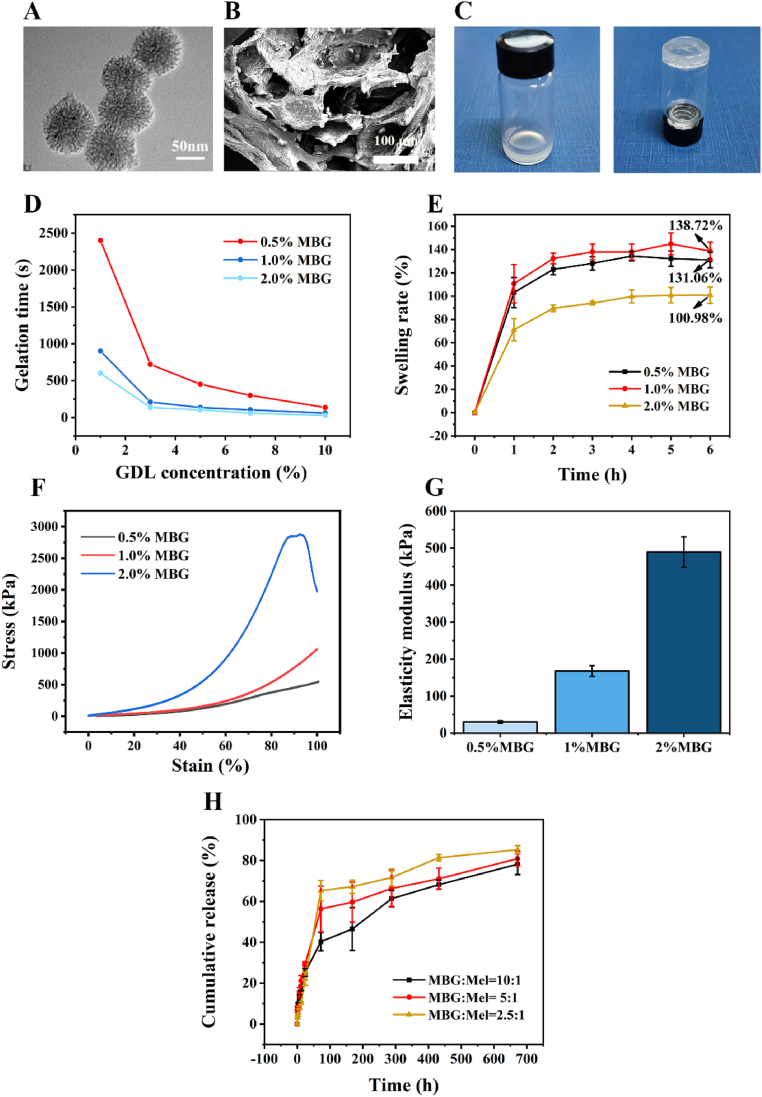
Characterization of mesoporous bioactive glass (MBG) and Mel-MBG/SA hydrogels. (A) The morphology of MBG nanoparticles, with a diameter of approximately 60 nm, is depicted in the Transmission Electron Microscopy (TEM) images, as evidenced by the accompanying scale bar of 50 nm. (B) Scanning electron microscope (SEM) image of the Mel-MBG/SA hydrogels (Scale bar: 100 μm). (C) Photos of the MBG/SA hydrogels before and after crosslinking. (D) Gelling time of hydrogels. (E) Swelling ratio of the hydrogels. (F) Stress-strain curves of different hydrogels upon tensile loading at a fixed strain rate. (G) Compression moduli of 0.5%, 1%, and 2% MBG/SA scaffolds. (H) The cumulative release rates of Mel in MBG/SA hydrogels with different Mel contents within 30 days.

In this study, we synthesized MBG nanoparticles using a microemulsion-assisted sol-gel method. TEM images revealed that the nanoparticles had uniform spherical and dendritic structures with diameters of ∼100 nm (Fig. 1A). This morphology closely resembled that of mesoporous silicon nanoparticles prepared using the similar method [46]. The mesoporous structure of MBG, characterized by its large specific surface area and high pore volume, enables it to offer ample space and active sites for the loading of Mel. When Mel-loaded MBG nanoparticles are exposed to an acidic environment, typically generated by the hydrolysis of GDL, they exhibit a rapid release of Ca^2+^ ions. These released ions serve as crosslinkers for SA macromolecules, leading to the formation of Mel-MBG/SA hydrogels. The microstructure of lyophilized Mel-MBG/SA hydrogels was examined by SEM, which showed the detailed microstructure of the hydrogels (Fig. 1B). The porous structure of the hydrogels could improve drug delivery efficiency, reduce the amount of degradation products, promote cell-cell and cell-matrix interactions, and provide an easy pathway for nutrient and metabolic waste transfer. It is worth noting that the addition of Mel-MBG to the SA suspension did not result in a significant gelation. However, with the addition of GDL, the Mel-MBG/SA hydrogels displayed a remarkable propensity for rapid gelation (Fig. 1C). This process involves three forms of cross-linking, namely, calcium ion cross-linking, physical cross-linking, and hydrogen cross-linking, which contribute to the gelation process. Increasing the concentrations of both MBG and GDL in the hydrogels resulted in shorter gelation times.

The properties of composite hydrogels with different concentrations of MBG and GDL were systematically determined. Hydrogels containing 1% and 2% MBG exhibited significantly shorter gelation times compared to those containing 0.5% MBG at the same GDL concentration. By adjusting the concentrations of MBG and GDL in the hydrogel formulation, the gelation time can be finely tuned to meet the specific requirements of various applications (Fig. 1D). In the swelling ratio test, the composite hydrogels gradually absorbed SBF, and all hydrogels reached swelling equilibrium after 24 h of SBF immersion. The swelling ratio of hydrogels containing 2% MBG was significantly lower than that of hydrogels containing 1% and 0.5% MBG at the same GDL concentration, indicating the better mechanical stability of the hydrogels with higher MBG concentrations (Fig. 1E). Previous studies have shown that mechanical properties are crucial for repairing medullary nucleus defects [47]. To further assess the mechanical properties of the Mel-MBG/SA hydrogels, compression tests were conducted. Given that these hydrogels are designed for IVD repair applications, the compressive mechanical behavior holds significant importance as a key parameter. Fig. 1F displays representative compressive stress-strain curves for the hydrogels, exhibiting a typical “J-shaped” curve. This distinctive curve shape closely resembles the mechanical behavior observed in biological tissues, including IVD. The compressive modulus of the hydrogels increased from approximately 0.5 MPa to a maximum strain of approximately 0.75 and 2.75 MPa (90%) at different MBG concentrations of 0.5%, 1%, and 2%. Additionally, the compressive strength of the hydrogels fell within the range of compressive strength values of human cancellous bone, which is approximately 0.15–13.7 MPa. Furthermore, the tensile strength of the hydrogels consistently increased with increasing MBG concentrations, and the hydrogel with 2% MBG exhibited a tensile strength of up to 500 KPa (Fig. 1G). Overall, these effects can enhance tissue regeneration and integration with the host in a synergistic manner. Consequently, the hydrogels containing 2% MBG and 3% GDL were selected for further experiments. The release behavior of Mel from the Mel-MBG/SA hydrogel was also investigated, and an initial burst of Mel release occurred during the first three days, followed by a relatively slow release. After 20 days, the accumulated amount of Mel released from the Mel-MBG/SA hydrogel was 80% (Fig. 1H). Based on these results, a formulation with an optimal ratio of GDL to MBG was identified for further studies.

In order to further elucidate the physico-chemical properties of the synthesized MBG, MBG/SA hydrogels, and Mel-MBG/SA hydrogels, a series of analytical techniques were employed, including XRD mapping, FT-IR, and ^13^C NMR characterization. Specifically, the ^13^C NMR analysis revealed the appearance of multiple chemical shift peaks corresponding to melatonin C element following the addition of Mel to the hydrogel (Fig. S1). Additionally, the XRD pattern of MBG exhibited a broadened characteristic diffraction peak of silicate bioactive glass within the 20–35° range, indicating the amorphous nature of the synthesized MBG powder (Fig. S2). When the concentration of Mel dopant in the hydrogel was limited, the absence of crystallization during system movement did not significantly impact the XRD pattern. However, as the Mel content increased, the lyophilized hydrogel exhibited crystalline peaks of Mel (2θ = 10.82, 16.34, 19.03 and 26.22), thereby confirming the successful incorporation of Mel into the system. In the context of FT-IR analysis, the presence of Mel within the hydrogel has minimal impact on the IR spectrum. Nonetheless, upon closer examination, it is apparent that the C-O-C stretching vibration peak, situated at approximately 1225 cm^−1^, undergoes broadening and red-shifting as the Mel concentration increases (Fig. S3). This phenomenon can be attributed to the IR absorption of pure melatonin's C-O-C stretching vibration, which is situated at 1212 cm^−1^ and differs from that of sodium alginate.

### 3.2 *In vitro* cytocompatibility

**Fig. 2:**
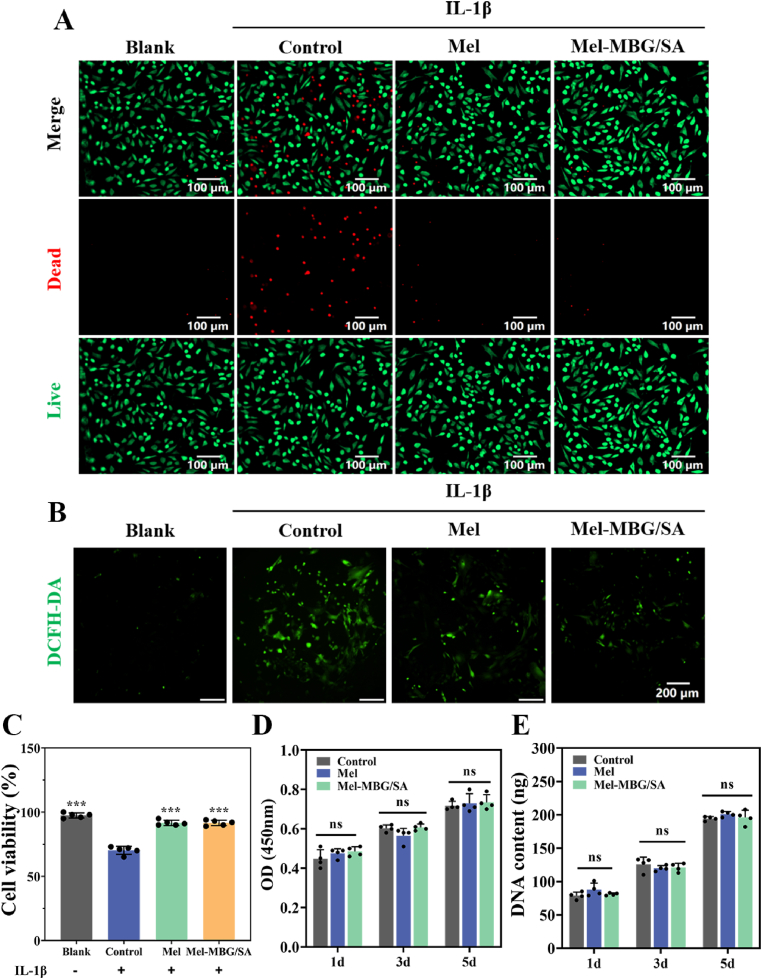
Cell proliferation and viability assay. (A) The viability of NPCs treated with different extracts for 24 h was evaluated by live/dead staining, scale bar = 100 μm (B) The reduction of oxidative stress in NPCs was monitored using a ROS probe (DCFH-DA) after different treatments, scale bar = 200 μm. (C) After IL-1β-induced inflammation or blank control, the viability of NPCs was assessed by CCK8 after 24 h of treatment with different extracts (n = 5). (D) The proliferation of NPCs was evaluated by the CCK8 test after treatment with different extracts for 1, 3 and 5 days (n = 5). (E) the DNA content of each group was calculated. Data are expressed as mean ± 95% confidence interval of n = 5 experiments. Statistically significant values are presented as * p < 0.05, **p < 0.01, and ***p < 0.001.

Biocompatibility is a vital prerequisite for the utilization of biomaterials in various applications. To evaluate the *in vitro* cytocompatibility of hydrogels, primary NPCs were isolated from IVDs. Initially, the hydrogel extract was added to rat NPCs, along with Mel solution, and the cell viability was determined by live/dead staining. Compared with the untreated control group, IL-1β-pretreated NPCs exhibited decreased cell viability and a higher number of dead cells represented by red fluorescence, suggesting that IL-1β could induce an oxidative stress injury. In contrast, when NPCs were treated with extracts of 50 μM melatonin and Mel-MBG/SA hydrogel for 24 h, only a minimal number of dead cells (as indicated by red fluorescence) were observed. Additionally, these treated cells exhibited almost no signs of toxicity (Fig. 2A). The findings indicate that Mel and Mel-MBG/SA possess the ability to mitigate oxidative stress damage caused by IL-1β and enhance cellular viability. Furthermore, statistical analysis of the percentage of surviving cells revealed significant disparities (p < 0.001) among the various treatments after 24 h in all other groups (cellular activity nearly 100%) relative to the inflammatory control group (70.20 ± 5.21%) (Fig. 2C). The results indicated that Mel-MBG/SA effectively inhibited the IL-1β-induced decrease in cell viability. This effect can be attributed to the continuous slow release of Mel provided by the Mel-MBG/SA hydrogel, in contrast to the rapidly degrading Mel. Additionally, CCK-8 assay was performed to evaluate the cytotoxicity of IL-1β-induced 50 μM Mel and Mel-MBG/SA hydrogels on induced chondrocytes. The viability of NPCs without IL-1β stimulation was unaffected by Mel and Mel-MBG/SA hydrogels compared to normal controls, indicating that Mel and Mel-MBG/SA hydrogels possess good cytocompatibility (Fig. 2D). Over a period of five days, the quantity of cells present in Mel and Mel-MBG/SA hydrogels exhibited a consistent increase, as evidenced by a significant rise in DNA content (Fig. 2E). These findings suggest that Mel-MBG/SA hydrogels possess favorable characteristics with respect to both cell viability and proliferation. The Supplementary Fig. S4 A and **B** illustrates the occurrence of cellular necrosis and skeletalization within 24 h of culturing on Mel-MBG/SA hydrogels. These results highlight that the cytotoxicity of both Mel and Mel-MBG/SA hydrogels is negligible.

To simulate the inflammatory response induced by oxidative stress in IDD, NPCs were treated with IL-1β to create an oxidative stress microenvironment *in vitro*, as mentioned earlier [48]. Next, the protective effects of Mel and Mel-MBG/SA hydrogels on these cells were examined. The changes in intracellular ROS were detected using the ROS indicator DCFH-DA, as shown in Fig. 2B. The results demonstrated that NPCs co-incubated with Mel or Mel-MBG/SA hydrogels had significantly lower green fluorescence compared to other control groups, indicating that Mel released by free or hydrogels can effectively alleviate IL-1β-induced oxidative stress *in vitro*.

### 3.3 *In vitro* IVD regeneration assessment

**Fig. 3:**
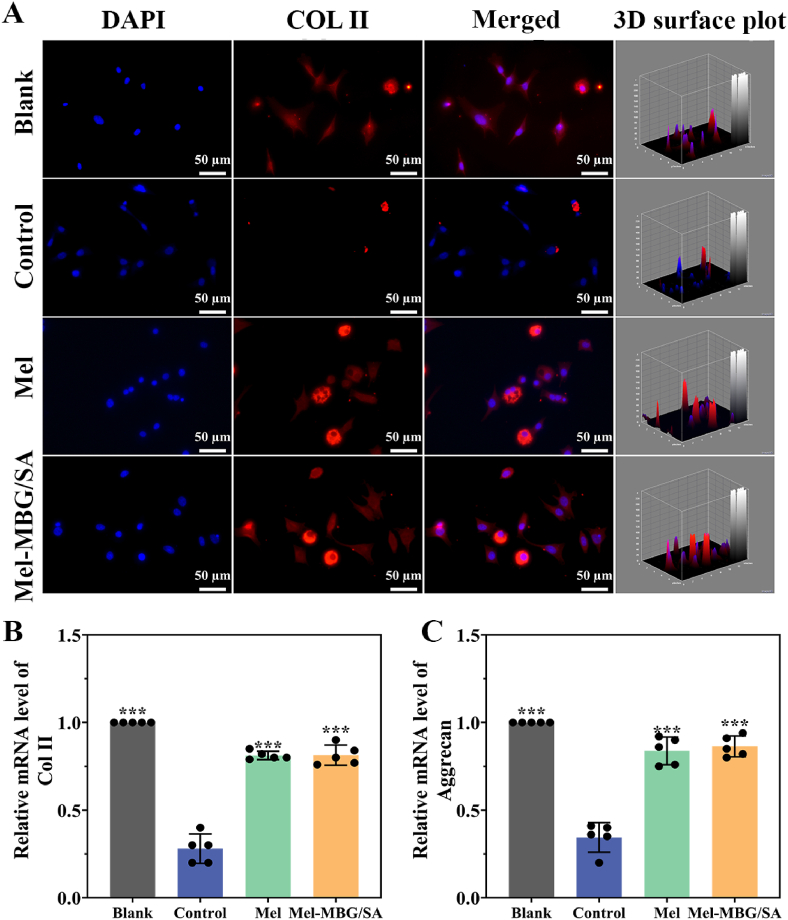
Mel-MBG/SA hydrogels enhance extracellular matrix synthesis and alleviate inflammatory response after IL-1β stimulation. (A) Immunofluorescence analysis of COL-II expression in NPCs under different treatments. (B-C) The semi-quantitative analysis of the mRNA expression levels of aggrecan and COL-II. Data are presented as means ± SD. *** indicates p < 0.001 in comparison with the control group, respectively. n = 5. ns: not significant.

To investigate the protective effect of Mel on IDD, an *in vitro* inflammatory environment was established by adding 5 ng/mL of IL-1β to the culture of NPCs, followed by co-culture of NPCs with Mel and Mel-MBG/SA hydrogels. Fig. 3A shows the representative type II collagen immunofluorescence staining after 24 h of co-culture. The control group exhibited significantly reduced fluorescence intensity due to the influence of inflammatory factors, indicating a decrease in the ECM synthesis capacity of NPCs in the inflammatory environment. In contrast, the Mel and Mel-MBG/SA groups demonstrated strong staining of type II collagen, suggesting that Mel release promotes the viability of NPCs by maintaining ECM synthesis.

The results of fluorescence microscopy and real-time PCR demonstrated that the expression levels of COL2A1 were significantly downregulated in the IL-1β stimulated groups (Control, Mel, and Mel-MBG/SA groups, as shown in Fig. 3A and **B**). However, a stronger fluorescent intensity of COL2A1 was observed in NPCs treated with melatonin (Mel and Mel-MBG/SA groups) compared to Control group (Fig. 3A). Moreover, Mel significantly upregulated the mRNA expression of COL2A1 in both Mel and Mel-MBG/SA groups compared to Control group (Fig. 3B). Additionally, the mRNA level of aggrecan was higher in both Mel and Mel-MBG/SA groups than in Control group (Fig. 3C). These findings demonstrate that Mel supplementation significantly increases the expression of nucleus pulposus phenotype-associated genes (collagen II and aggrecan) in NPCs.

**Fig. 4:**
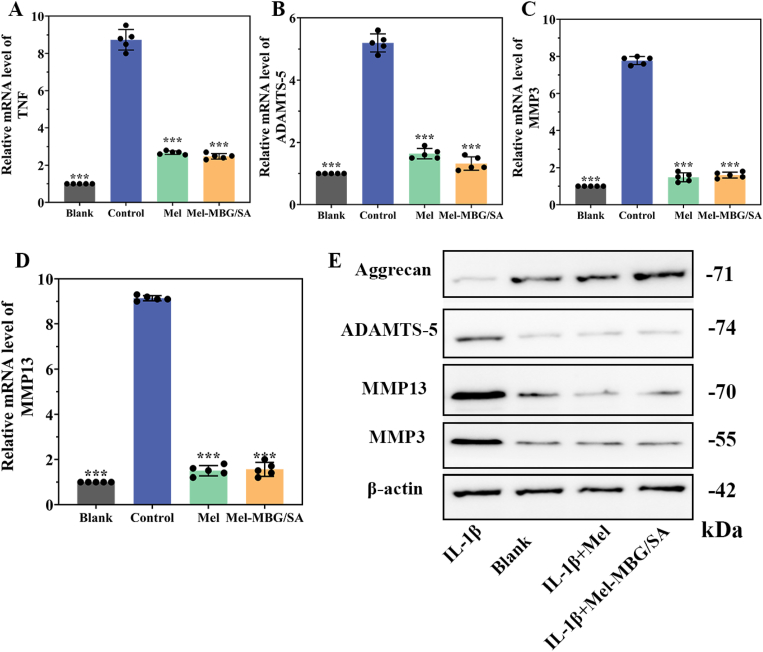
Effect of Mel-MBG/SA on inflammation and ECM homeostasis. After pretreatment of NP cells with IL-1β for 3 days, (A) Gene expression of the major inflammatory factor TNF in the blank, control, Mel and Mel-MBG/SA groups (n = 5), (B–D) qRT-PCR results of the relative mRNA levels of the major ECM degrading enzymes ADAMTS-5, MMP-3, and MMP-13 (n = 5). (E) Protein levels of NP cell matrix components and ECM degrading enzymes, including aggrecan, ADAMTS-5, MMP-3 and MMP-13 by western blot after 3 days of IL-1β treatment. NP cells without IL-1β pretreatment were set as blank group and NP cells with IL-1β pretreatment only were set as control group. Data are presented as means ± SD. *** indicates p < 0.001 in comparison with the control group, respectively.

To investigate the effect of Mel on IL-1β-induced inflammation, the mRNA expression levels of several inflammatory genes, including TNF, ADAMTS5, MMP-3, and MMP-13, were analyzed by real-time PCR assays (Fig. 4A and B, C, D). The results showed that the expression levels of these inflammatory genes were significantly upregulated after IL-1β induction. However, after treatment with Mel, the expression levels of TNF, ADAMTS5, MMP-3, and MMP-13 were significantly down-regulated in both Mel and Mel-MBG/SA groups, indicating that inflammation was significantly suppressed in these groups. Similar to the RT-PCR results, after Mel only or Mel-MBG/SA hydrogel addition, Western blot results showed a significant increase in aggrecan expression and a decrease in MMP3, MMP13, and ADAMTS-5 expression in IL-1β-treated NP cells (Fig. 2E). These findings suggest that Mel and Mel-MBG/SA hydrogels can effectively inhibit IL-1β-mediated inflammation and oxidative stress, promote the activity of NPCs, upregulate the expression of ECM molecules, and promote the repair and regeneration of IDD. However, the molecular mechanisms underlying these effects require further investigation.

### 3.4 *In vivo* therapeutic effect

**Fig. 5:**
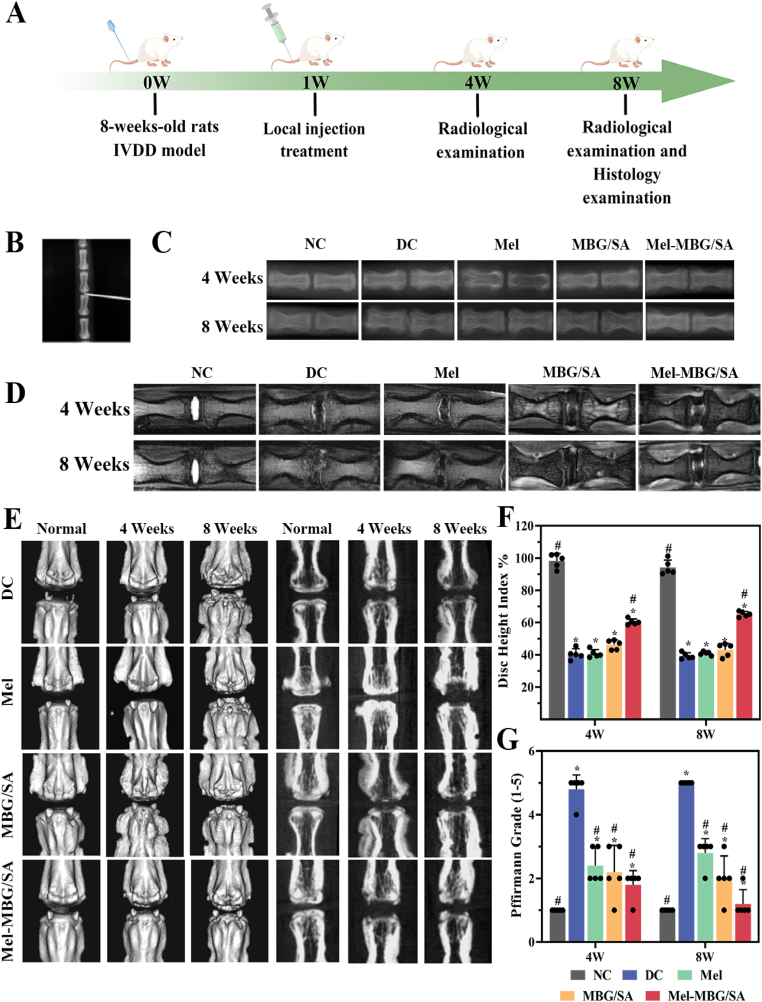
Radiological data of animal experiments by imaging system. (A) Experimental design of rat IDD by Figdraw. (B) Establishment of the caudal vertebrae puncture model in rats is depicted. (C) Representative X-ray images of the caudal vertebrae of rats at 4 and 8 weeks. (D) Representative MRI images of the caudal vertebrae of rats. (E) Micro-CT examination of the rat intervertebral discs 4 weeks and 8 weeks after treatment. (F) Quantitative analysis of DHI changes in different groups at 4 and 8 weeks after molding. (G) Quantitative analysis of MRI grade changes in different groups at 4 and 8 weeks after molding. (Data are presented as means ± SD. n = 5, # and * indicate P < 0.001 in comparison with the DC and NC groups, respectively).

A puncture-induced rat model of IDD (Fig. 5A) was used to evaluate the therapeutic effect of Mel-MBG/SA hydrogels. Rat IVDs were administered injections of Mel or hydrogels through a 26G needle, while the NC group remained unpunctured, and the DC group received an equivalent amount of PBS. The localization of the needle was conducted with the assistance of x-ray guidance (Fig. 5B). Radiological assessment of the disc height index (DHI) in the five groups showed that at 4 weeks post-surgery, the DHI values of Mel-MBG/SA group were similar to those of normal control (NC) group, but significantly higher than those of Mel and DC groups. Meanwhile, the DHI values observed in the MBG/SA group exhibited a marginal increase compared to the inflammatory degenerative disease control group (DC group), albeit lacking statistical significance. At the 8-week postoperative mark, the DHI exhibited a greater value in the Mel-MBG/SA group compared to the remaining groups. No statistically significant distinction was observed between the Mel group and the DC and MBG/SA groups (Fig. 5C, E, F). These results suggest that Mel-MBG/SA significantly delays the puncture-induced disc height reduction, while Mel alone has little therapeutic effect.

MRI analysis provides information about the water content of IVDs, and serves as a valuable indicator in diagnosing IDD. To quantify the MRI results, the Pfirrmann Grade classification method [49] was employed to grade the MRI images into five categories based on disc structure and signal intensity. Grade I represents a normal disc structure with a bright hyperintense white signal intensity and normal disc height, while Grade V represents an inhomogeneous structure with a hypointense black signal intensity. As shown in Fig. 5D, G, the T2-weighted MR image of IVDs in Mel-MBG/SA group demonstrated stronger intensities compared to the DC and Mel groups, as well as the MBG/SA group. The Mel-MBG/SA group showed a clear distinction between the nucleus and anulus, as well as a normal disc height with minimal horizontal gray bands. However, the groups consisting of DC and Mel, as well as the MBG/SA group, showed lost distinction between the nucleus and anulus. At the 4 and 8-week intervals, the Pfirrmann grade exhibited a statistically significant decrease in the Mel-MBG/SA group compared to the DC, Mel, and MBG/SA groups. This finding suggests that the Mel-MBG/SA hydrogel effectively stimulates ECM production, impedes the degenerative process, and preserves the structural integrity of the IVD.

**Fig. 6:**
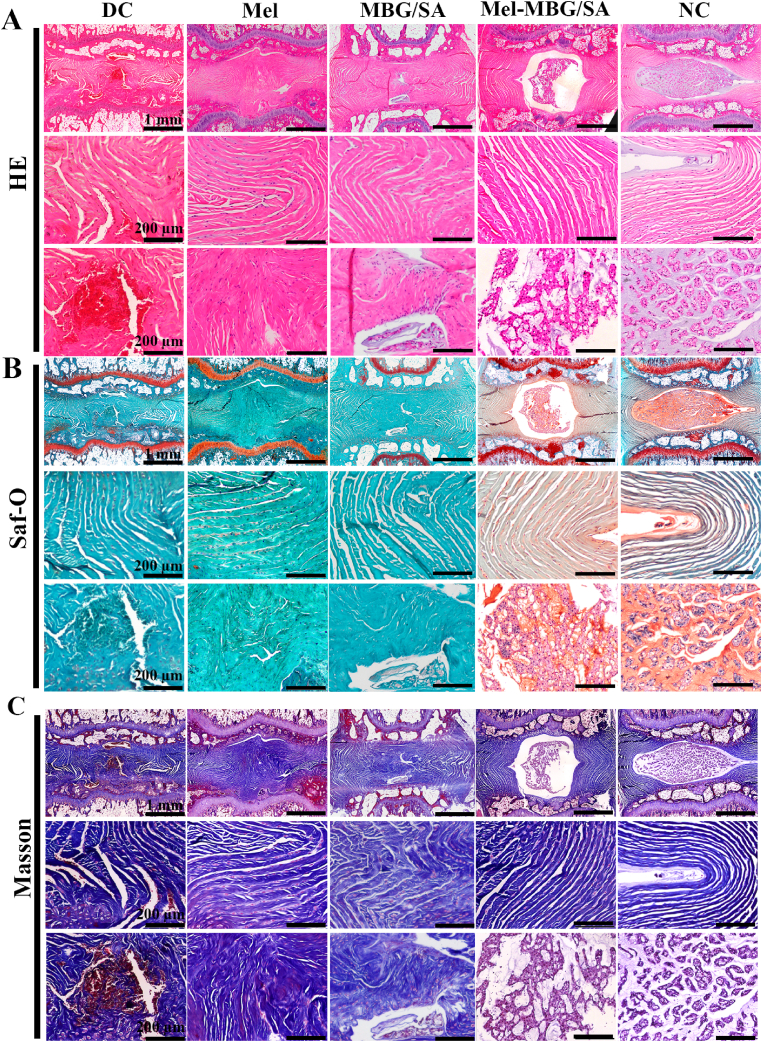

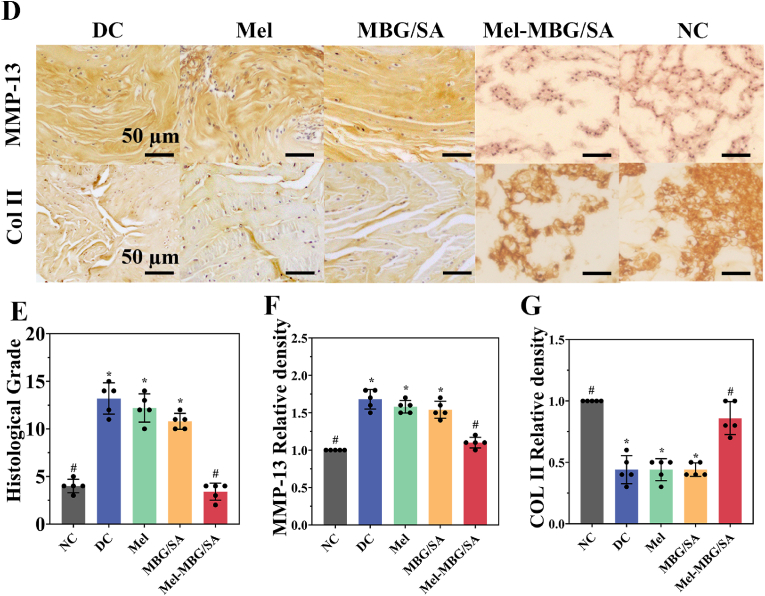
HE staining, Saf-O staining, Masson Trichrome (positive for collagen in the extracellular matrix) and immunohistochemical staining (positive for type II collagen and MMP13) to assess the extent of nucleus pulposus repair and degeneration in vivo. (A–B) HE and Saf-O staining in the DC and Mel and MBG/SA groups showed significant defects in the nucleus pulposus region, while the nucleus pulposus region was replaced by fibrous tissue. In contrast, the nucleus pulposus region was intact in the NC and Mel-MBG/SA groups. In the intact discs of the NC and Mel-MBG/SA groups, (C–D) Masson staining and MMP13 were negative in the nucleus pulposus region. In the punctured DC and Mel and MBG/SA groups, the nucleus pulposus region was positive for Masson staining and MMP13 staining, whereas type II collagen exhibited an opposite trend. (E) Changes in the histological scores of five groups at 4 and 8 weeks after surgery. (F–G) Quantitative analysis of type II collagen and MMP13 immunohistochemistry. (Data are presented as means ± SD. n = 5, # and * indicate P < 0.001 in comparison with the DC and NC groups, respectively).

The histological grading system developed by Masuda et al. [45] was used to evaluate the degenerative changes in IVDs. This grading system assigned a grade of 1, 2, or 3 to four parameters: the annulus fibrosus, the border between the annulus fibrosus and the nucleus pulposus, the cellularity of the nucleus pulposus, and the matrix of the nucleus pulposus. By summing up the grades for each parameter, a total grade was obtained, which ranged from 4 to 12. A higher total grade indicated more severe degeneration, with 12 representing the most severe level of degeneration. Our results showed that the total histological grade of Mel-MBG/SA group (3.4 ± 0.90) was significantly lower than those of Mel group (12.2 ± 1.48) and MBG/SA group (10.8 ± 0.84) and DC groups (13.2 ± 1.64) (Fig. 6E). In order to examine the therapeutic mechanism of Mel-MBG/SA, the immunohistochemical staining technique was employed to assess the expression levels of MMP-13 and type II collagen. The nucleus pulposus region exhibited a lack of MMP-13 staining and enhanced staining for type II collagen in both the NC and Mel-MBG/SA groups (Fig. 6D). Conversely, the DC and Mel groups, as well as the MBG/SA group, demonstrated heightened immunoreactivity for MMP-13 and diminished staining for type II collagen. The results of this study indicate that the administration of Mel-MBG/SA effectively reduces inflammation induced by needle puncture in vivo. Analysis of MMP-13 protein levels revealed significantly elevated levels in the Mel and DC groups, as well as the MBG/SA group, when compared to the NC group and the Mel-MBG/SA group. Conversely, analysis of type II collagen levels demonstrated lower levels in the Mel, DC, and MBG/SA groups compared to the NC and Mel-MBG/SA groups (Fig. 6F and G).

## 4 Discussion

IDD can result in pain, neurological deficits, and disability among patients [50]. Regrettably, the existing treatments available for disc degeneration merely focus on mitigating the painful symptoms and do not provide a definitive solution for addressing the underlying condition itself [51]. Indeed, tissue engineering and regenerative medicine have emerged as more promising avenues for relieving low back pain and restoring the function of degenerated discs [52]. Researchers are currently developing biomaterials specifically for the treatment of IDD. These biomaterials can be categorized into two primary types: those with high mechanical strength and those with flexibility and softness.

Examples of biomaterials with high mechanical strength include organic polymers and metallic materials. These biomaterials are commonly utilized in surgical therapies for IDD to immobilize vertebral segments, ensuring their stability. However, it is worth noting that while biomaterials with high mechanical strength can provide stability to the affected spinal segments, their extreme or excessive strength can potentially have negative consequences. One such consequence is the potential for cartilage endplate damage, which can lead to secondary degeneration of adjacent disc segments [53]. Indeed, biomaterials with flexibility and softness, such as hydrogels, offer distinct advantages for the treatment of IDD. Hydrogels, in particular, are promising candidates for nucleus pulposus tissue engineering. They possess properties that closely resemble the natural nucleus pulposus tissue, including hydrophilicity and rheological characteristics [54]. Although hydrogels have shown potential for restoring disc height and promoting self-renewal of NPCs [55], their clinical applications can be hindered by their poor mechanical properties.

Various methods have been employed to improve the mechanical properties of hydrogels. However, it can be a challenging task to enhance the mechanical strength while simultaneously preserving all the desirable properties. It has been reported that the incorporation of MBG into polymer hydrogels can lead to improvements in their mechanical properties, alongside promoting the healing of bone and cartilage [56]. SA, being a natural organic polymer with remarkable biocompatibility, exhibits facile cross-linking with diverse ions at ambient temperature, thereby facilitating rapid gel formation, which is highly compatible with MBG's multi-ionic constituents. The amalgamation of 10.13039/100010493MBG and SA is thus highly appropriate, as it takes only a few minutes at room temperature to achieve complete curing and provide support upon injection into the disc defect site.

The melatonin receptor consists of seven transmembrane proteins and two major isoforms, MT1 and MT2. Li et al. identified the presence of MT1 and MT2 receptors in human NP cells for the first time [57]. The analysis of receptor blockade suggests that the melatonin-induced ECM remodeling in NP cells is mediated by its membrane receptor (MT1/2). However, due to the non-specificity of receptor antagonists for MT1/2, it is not possible to determine the specific role of MT1 or MT2 in this process. Qiu et al. demonstrated that melatonin can activate the MT1 receptor to reverse TNF-induced metabolic changes in NP cells [58]. Furthermore, melatonin-induced upregulation of YAP leads to increased expression of I-κBα and subsequent inhibition of the NF-κB pathway activation induced by tumor necrosis factor, thereby effectively suppressing the catabolic processes in NP cells. Moreover, Zhou et al. established that the administration of melatonin *in vitro* can regulate the homeostasis of extracellular matrix in chondrocytes affected by osteoarthritis by specifically targeting MT2 receptors [59]. By capitalizing on the inherent presence of MT1/2 receptors on NP cells, we incorporated melatonin into the hydrogel to enhance the therapeutic efficacy in vivo.

In this study, we focused on three specific properties: mechanical properties, injectability, and anti-inflammatory effects, and Mel-laden mechanically adjustable MBG composite SA hydrogels were used to meet these requirements. A series of experiments were conducted to optimize the mechanical properties of Mel-MBG/SA hydrogels, evaluate the physical and biological properties of these hydrogels in relation to their potential for nucleus pulposus regeneration, and determine the most suitable preparation conditions for effective nucleus pulposus regeneration.

A Mel-loaded hydrogel was fabricated in combination with SA and MBG nanoparticles. SA acts as a drug delivery vehicle that provides an extracellular matrix function similar to native nucleus pulposus, while MBG functions as an enhancer to improve the mechanical properties. The results showed that Mel-MBG/SA hydrogels at different MBG concentrations of 0.5%, 1%, and 2% increased the hydrogel's compressive modulus from ∼0.5 MPa to a maximum strain of ∼0.75 and ∼2.75 MPa (∼90%). The compressive load experienced by natural discs typically ranges from 0.1 to 0.9 MPa [60,61], indicating the mechanical requirements for a suitable hydrogel in the disc environment. In this study, the hydrogel with a 2% concentration of MBG was found to be sufficient in adapting to the disc environment. To the best of our knowledge, this study represents the first systematic evaluation of the feasibility of MBG-doped SA hydrogels as scaffolds for nucleus pulposus regeneration.

Although our study have yielded promising results regarding the potential use of Mel-MBG/SA hydrogels for nucleus pulposus regeneration, there are several limitations that should be taken into consideration. One important limitation of our study is the exclusive use of female rats to collect tissue samples. This approach may have introduced a potential gender bias into our findings. Furthermore, the elderly demographic is commonly regarded as a focal group for disc degeneration. The utilization of rats in our animal experimentation was limited to those approximately 8 weeks old, as acquiring older rats proved challenging. The heightened regenerative potential of juvenile rats in contrast to their elderly counterparts warrants consideration. Consequently, the aging rat model may prove more appropriate in emulating the disc milieu of the elderly. Another limitation of our study is the relatively short duration of the cell viability tests conducted. The chosen duration might not have provided a complete representation of the long-term effects of Mel-MBG/SA hydrogels on cell viability. Lastly, it is important to note that the rat model of IDD generated through caudal disc puncturing may exhibit biological and biomechanical differences compared to human IDD. Hence, the induction of caudal disc inflammation in rats through surgical needling fails to entirely replicate the clinical manifestation of human disc degeneration. While mechanical manipulation or direct puncture-induced mouse models of IVD degeneration exhibit degenerative alterations at a relatively rapid pace, genetic models may provide a more accurate representation of IVD degeneration that occurs gradually with aging. Despite these limitations, our study provides valuable insights into the potential of Mel-MBG/SA hydrogels as a promising treatment option for IDD, and highlights the importance of continued exploration and research into innovative solutions for this prevalent and debilitating condition.

## 5 Conclusion

In summary, a novel Mel-MBG/SA hydrogel with integrated mechanical properties and drug release functions was designed and fabricated. By reinforcing SA hydrogels with optimized composition and architecture using MBG, we successfully developed a hybrid hydrogel that exhibits desirable physical and mechanical properties comparable to those of natural load-bearing IVD. The hydrogel exhibits compressive loading capabilities ranging from 0.75 to 2.75 MPa, which aligns with the compressive load range of natural intervertebral discs, spanning from 0.1 to 0.9 MPa [[61], [62], [63]]. The MBG/SA hydrogel offers a unique hybrid system that not only possesses desirable physical and mechanical properties but also provides sustained release of Mel, thereby attenuating IL-1β-induced oxidative stress and reducing inflammation associated with IDD pathology. Additionally, our study demonstrates that this delivery system can significantly inhibit inflammation in a rat caudal model. This breakthrough finding highlights the potential of our approach in guiding tissue regeneration by modulating the inflammatory environment in conjunction with the mechanical characteristics of the material.

## Credit author statement

Ruibang Wu: Investigation, Methodology, Validation, Software, Visualization. Leizhen Huang: Conceptualization, Investigation, Methodology, Validation, Software, Writing – original draft. Qinghong Xia: Investigation, Methodology, Software, Visualization. Zheng Liu: Investigation, Methodology, Software. Yong Huang: Investigation, Methodology. Yulin Jiang: Investigation, Methodology. Juehan Wang: Methodology, Software. Hong Ding: Investigation, Methodology. Ce Zhu: Methodology, Software. Yueming Song: Funding acquisition, Project administration, Resources, Supervision, Validation, Visualization, Writing – review & editing. Limin Liu: Funding acquisition, Project administration, Resources, Supervision, Validation, Visualization. Li Zhang: Funding acquisition, Project administration, Resources, Supervision, Validation, Visualization, Writing – review & editing. Ganjun Feng: Funding acquisition, Project administration, Resources, Supervision, Validation, Visualization, Writing – review & editing.

## Funding sources

This research was funded by the 10.13039/501100001809National Natural Science Foundation of China (No.82272546, 82172495, 82072434, 81871772, 81772397) and the 10.13039/100012542Sichuan Science and Technology Program (2021YFS0218, 2022ZDZX0029).

## Declaration of competing interest

The authors declare that they have no known competing financial interests or personal relationships that could have appeared to influence the work reported in this paper.

## Data Availability

Data will be made available on request.

## List of abbreviations

IDD: iIntervertebral disc degeneration
IVD: intervertebral disc
NP: nucleus pulposus
AF: annulus fibrosus
CEP: cartilaginous endplate
Mel: melatonin
ECM: extracellular matrix
TNF: tumor necrosis factor
IL-1β: interleukin-1β
IL-6: interleukin-6
INOS: inducible nitric oxide
COX-2: cyclooxygenase-2
NO: nitric oxide
PGE2: prostaglandin E2
MSC: mesenchymal stem cell
ROS: reactive oxygen species
SA: sodium alginate
BG: bioactive glass
MBG: mesoporous bioactive glass
GDL: glucono-δ-lactone
CTAB: Cetyltrimethylammonium bromide
TEOS: Tetraethyl orthosilicate
SBF: Simulated Body Fluid
DMEM: Dulbecco's Modified Eagle Medium
SD: Sprague-Dawley
BSA: bovine serum albumin
GAPDH: glyceraldehyde-3-phosphate dehydrogenase
NC: normal control
DC: degeneration control
μCT: microcomputed tomography
DHI
disc height index
EDTA: ethylenediaminetetraacetic acid
HE: hematoxylin-eosin
Saf-O: Safranin O/fast green
