# Supplementary material for: Injectable mesoporous bioactive glass/sodium alginate hydrogel loaded with melatonin for intervertebral disc regeneration
Source: Mater Today Bio. 2023 Jul 17;22:100731. doi: 10.1016/j.mtbio.2023.100731 (PMC10393589; doi:10.1016/j.mtbio.2023.100731)
Supplement: Multimedia component 1 [file mmc1.docx]

**Injectable Mesoporous Bioactive Glass/Sodium Alginate Hydrogel Loaded with Melatonin for Intervertebral Disc Regeneration**

Ruibang Wu^1,#^, Leizhen Huang^1,#^, Qinghong Xia^3,#^,Zheng Liu^2^ , Yong Huang^1^, Yulin Jiang^2^ , Juehan Wang^1^，Hong Ding^1^, Ce Zhu^1^, Yueming Song, Limin Liu^1^*, Li Zhang^2^*, and Ganjun Feng^1^*

1. Department of Orthopedic Surgery and Orthopedic Research Institute, West China Hospital, Sichuan University, Chengdu 610041, Sichuan, China.
2. Analytical and Testing Center, Sichuan University, Chengdu 610065, China.
3. Operating Room of Anesthesia Surgery Center, West China Hospital, Sichuan University / West China School of Nursing, Sichuan

*** To whom correspondence should be addressed:**

Limin Liu, E-mail: liulimin_spine@163.com;

Li Zhang, E-mail: [nic1976@scu.edu.cn](mailto:nic1976@scu.edu.cn)

Ganjun Feng, E-mail: [gjfenghx@163.com](mailto:gjfenghx@163.com)

**^#^ The three authors contributed equally to the work.**

**Supplementary information**

| **Name** | **Gene ID** | **Primer** | **Sequences** |
| --- | --- | --- | --- |
| Collagen Ⅱ | 25412 | Forward  Reverse | CAC GCA TGA GCC GAA GCT A  GGG TTT CCA CGT CTC ACC A |
| Aggrecan | 58968 | Forward  Reverse | CAC TGT CAA AGC ACC ATG CC  TAG GCT GGC TCC CAT TCA GT |
| MMP13 | 171052 | Forward  Reverse | CAA GCA GTT CCA AAG GCT ACA  TAG GGC TGG GTC ACA CTT CT |
| ADAMTS5 | 304135 | Forward  Reverse | ATG CAG CCA TCC TGT TCA CC  AAG GCC AAG TAG ATG CCC AAT TT |
| IL-6 | 451958166 | Forward  Reverse | CCG GAG AGG AGA CTT CAC AG  TCC ACG ATT TCC CAG AGA AC |
| β-actin | 55574 | Forward  Reverse | AGC CAT GTA CGT AGC CAT CC  CTC TCA GCT GTG GTG GTG AA |

***Table S1.*** *Real-time PCR primer sequences used in this study.*

*All primers are designed for rat genes.*

**
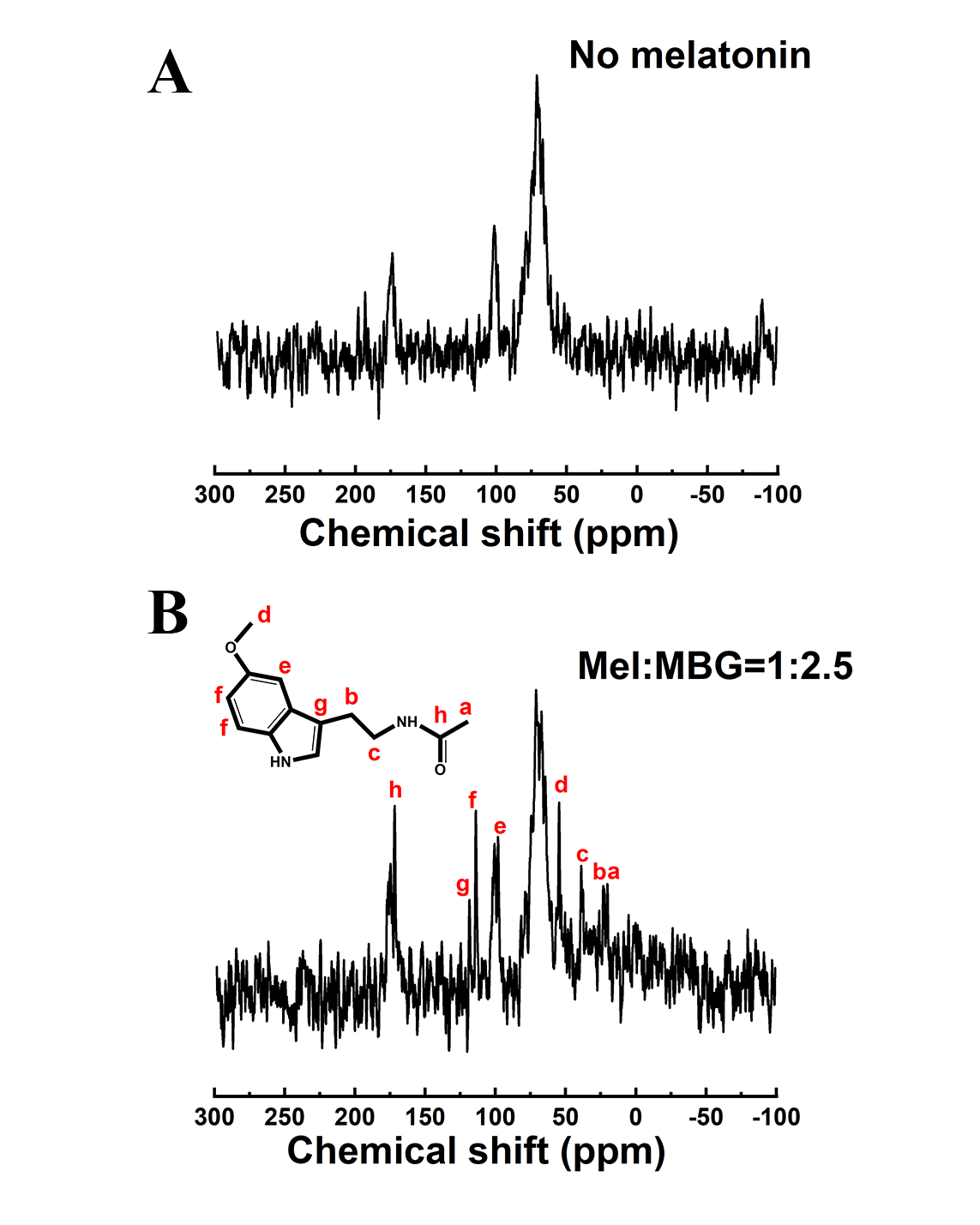
**

***Figure S1.*** *^13^C-NMR spectra of (A) MBG/SA hydrogel and (B) Mel-MBG/SA hydrogel with a mass fraction ratio of 1:2.5 of Mel to MBG.*

**
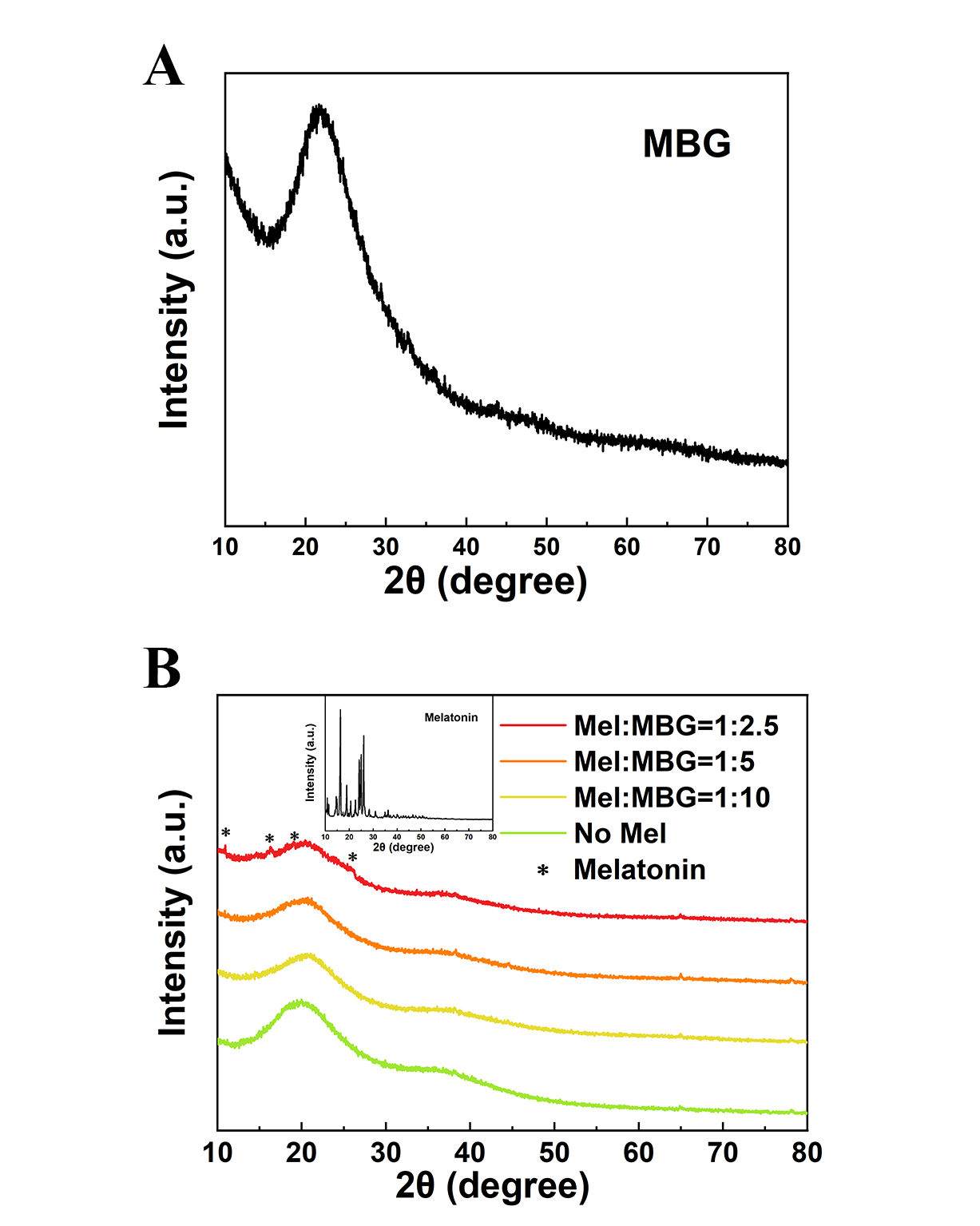
**

***Figure S2.*** *X-ray diffraction (XRD) patterns of (A) MBG and (B) MBG/SA hydrogels, as well as Mel-MBG/SA hydrogels with the mass fraction ratio of Mel to MBG (1:2.5, 1:5, and 1:10).*

**
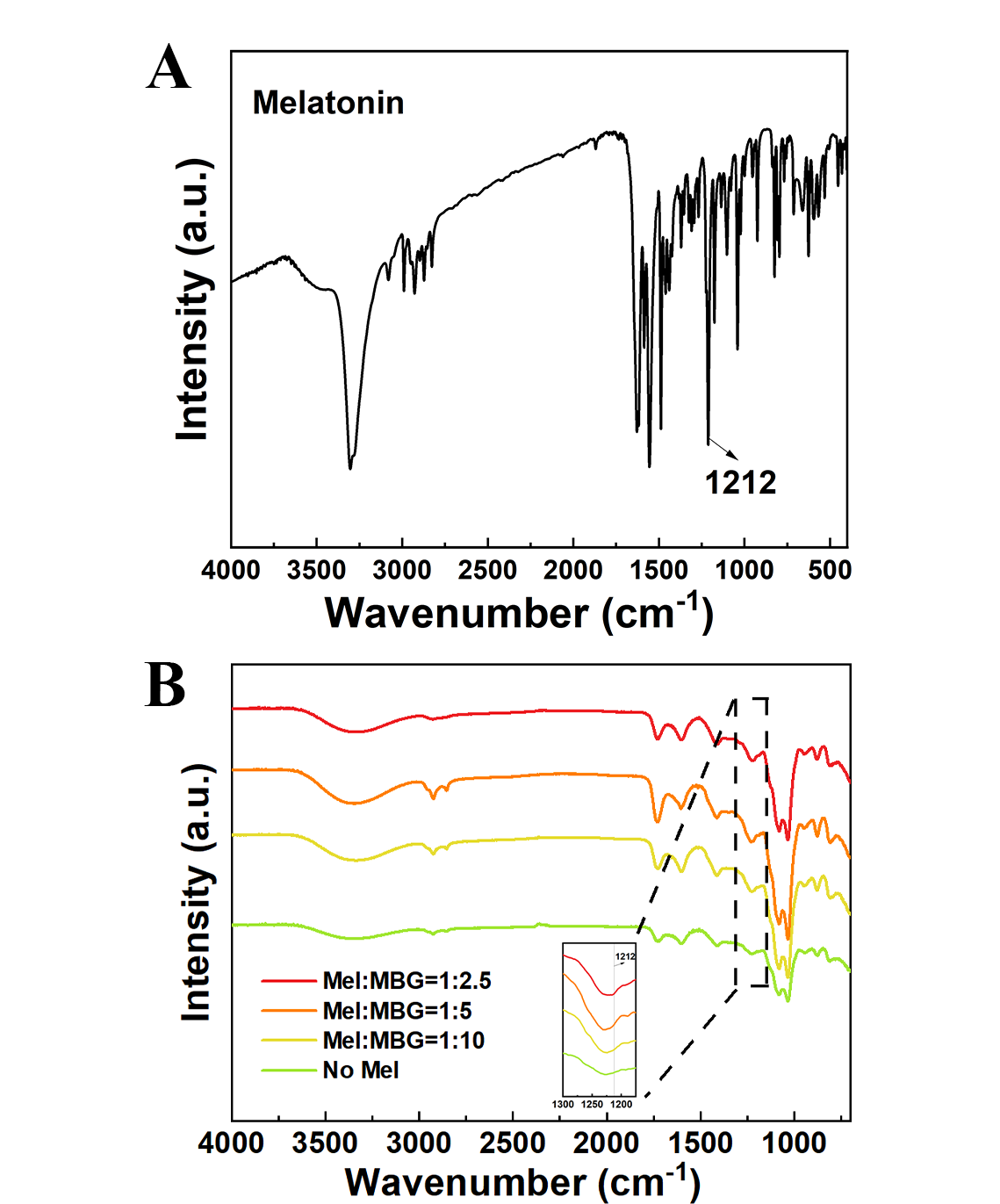
**

***Figure S3.*** *FT-IR spectra of (A) Melatonin and (B) MBG/SA hydrogels, as well as Mel-MBG/SA hydrogels with the mass fraction ratio of Mel to MBG (1:2.5, 1:5, and 1:10).*

**
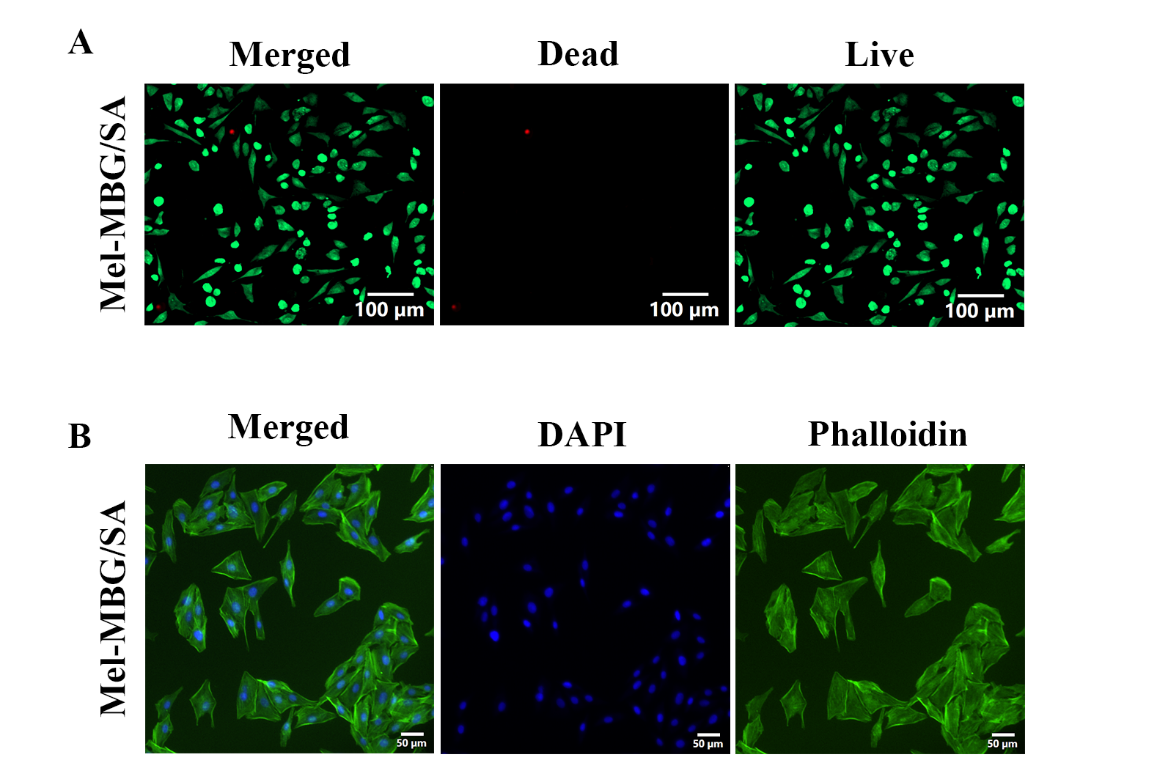
**

***Figure S4.*** *(A) Live-dead fluorescence imaging of NP cells after 24 h of culture on Mel-MBG/SA hydrogels and (B) cytoskeletal fluorescence staining (green represents cytoskeleton; blue represents nucleus).*
